# Mechanisms of ultrafine particle-induced respiratory health effects

**DOI:** 10.1038/s12276-020-0394-0

**Published:** 2020-03-17

**Authors:** George D. Leikauf, Sang-Heon Kim, An-Soo Jang

**Affiliations:** 1 Department of Environmental and Occupational Health, University of Pittsburgh, Pittsburgh, USA; 2 Department of Internal Medicine, Hanyang University, Seoul, Republic of Korea; 3 Department of Internal Medicine, Soonchunhyang University Bucheon Hospital, Bucheon, Republic of Korea

**Keywords:** Stress signalling, Diagnostic markers

## Abstract

Particulate matter (PM) is the principal component of air pollution. PM includes a range of particle sizes, such as coarse, fine, and ultrafine particles. Particles that are <100 nm in diameter are defined as ultrafine particles (UFPs). UFPs are found to a large extent in urban air as both singlet and aggregated particles. UFPs are classified into two major categories based on their source. Typically, UFPs are incidentally generated in the environment, often as byproducts of fossil fuel combustion, condensation of semivolatile substances or industrial emissions, whereas nanoparticles are manufactured through controlled engineering processes. The primary exposure mechanism of PM is inhalation. Inhalation of PM exacerbates respiratory symptoms in patients with chronic airway diseases, but the mechanisms underlying this response remain unclear. This review offers insights into the mechanisms by which particles, including UFPs, influence airway inflammation and discusses several mechanisms that may explain the relationship between particulate air pollutants and human health, particularly respiratory health. Understanding the mechanisms of PM-mediated lung injury will enhance efforts to protect at-risk individuals from the harmful health effects of air pollutants.

## Respiratory disease: Effects of airborne ultrafine particles

The ultrafine particles (UFPs) in air pollution aggravate respiratory illnesses via oxidative stress and inflammation, according to a review by South Korean and US researchers. Defined as particles less than 100 nm in diameter, UFPs are about 1/1000^th^ the thickness of a human hair. Inhaling UFPs is known to aggravate respiratory illnesses such as asthma, but the mechanisms remain poorly understood. An-Soo Jang at Soonchunhyang University Bucheon Hospital, Korea and co-workers have reviewed the evidence on how UFPs harm the airways. They report that UFPs are readily deposited in the lungs, where they trigger production of reactive oxygen species, highly reactive molecules that cause cellular and DNA damage. UFPs also provoke airway inflammation, and long-term exposure can scar the lungs. These results will aid in protecting at-risk individuals from the harmful effects of air pollution.

## Introduction

Particulate matter (PM) is the principal component of indoor and outdoor air pollution. PM includes a range of particle sizes, such as coarse, fine, and ultrafine particles. PM is a complex mixture of materials with a carbonaceous core and associated materials such as organic compounds, acids, and fine metal particles^1–3^. Particles that are <100 nm in diameter are defined as ultrafine particles (UFPs). UFPs are found to a large extent in urban air as both singlet and aggregated particles^4^.

UFPs are classified into two major categories based on their source. UFP typically refers to particles that are incidentally generated in the environment, often as byproducts of fossil fuel combustion, condensation of semivolatile substances or industrial emissions, whereas nanoparticles are manufactured through controlled engineering processes^4^.

The physical properties of PM, including the mass, surface area, and number/size/distribution of particles, as well as their physical state, influence respiratory health in different ways^2^. The primary exposure mechanism of PM is inhalation^2^. Inhalation of PM exacerbates respiratory symptoms in patients with chronic airway disease, but the mechanisms underlying this response remain unclear.

This review focuses on the adverse effects of exposure to ambient PM air pollution on the exacerbation, progression, and development of respiratory diseases such as asthma and chronic obstructive pulmonary disease (COPD). Of note, although air quality is improving in the US, UK, and other countries, the association of PM and COPD with asthma persists. For example, Hopke et al.^5^ compared the rate of COPD hospitalizations and emergency department visits in New York State before, during, and after the 2008 economic recession. The rate of asthma-related emergency department visits and COPD-related hospitalizations that were associated with each interquartile range increase in the concentration of ambient PM_2.5_ (PM that is <2.5 µm in diameter) was higher after the recession (2014–2016) than during (2008–2013) or before (2005–2007) it. For example, each 6.8 μg/m^3^ increase in PM_2.5_ on the same day was associated with 0.4%, 0.3%, and 2.7% increases in the rate of asthma-related emergency department visits before, during, and after the time period, respectively, suggesting that the same mass concentration of PM_2.5_ was more toxic after the recession.

Similarly, Doiron et al.^6^ used UK Biobank data on 3 03, 887 individuals aged 40–69 years, with complete covariate data and valid lung function measures. Cross-sectional analyses examined associations between land use regression-based estimates of particulate matter [PM_2.5_ and PM_10_ (PM that is less than 10 µm in diameter)] concentrations with forced expiratory volume in 1 s (FEV_1_), forced vital capacity (FVC), the FEV_1_/FVC ratio and COPD (FEV_1_/FVC < lower limit of normal). A 5 µg/m^3^ increase in PM_2.5_ concentration was associated with reduced FEV_1_ and FVC. COPD prevalence was associated with increased concentrations of PM_2.5_ (OR 1.52) and PM_10_ (OR 1.08) per 5 µg/m^3^. Robust associations with lung function were observed for males, individuals from lower-income households, and “at-risk” occupations, and increased COPD associations were observed for obese, lower-income, and non-asthmatic participants. Thus, ambient air pollution remains associated with reduced lung function and increased COPD prevalence.

This review offers insights into the mechanisms by which particles influence airway inflammation and discusses several mechanisms that may explain the relationship between particulate air pollutants and human health, particularly respiratory health. PM induces oxidative stress and inflammation, thereby stimulating innate and acquired immune responses in laboratory animals and humans. Understanding the mechanisms of PM-induced lung injury will enhance efforts to protect at-risk individuals from the harmful health effects of air pollutants.

## Mechanisms of UFP-induced health effects

**Fig. 1:**
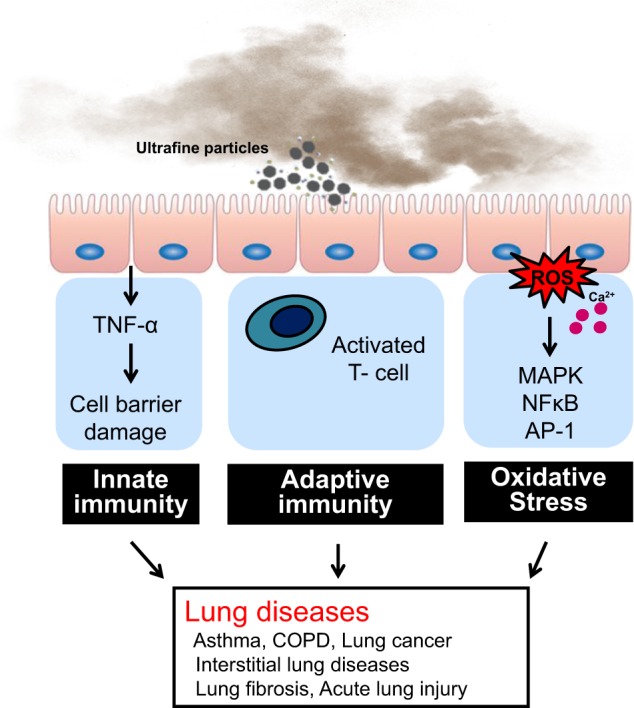
The proposed mechanism of ultrafine particle (UFP)-induced lung diseases. PM causes the activation of oxidative stress and reactive oxygen species, innate immunity, adaptive immunity, and other mechanisms, leading to the development and exacerbation of respiratory diseases such as bronchial asthma, COPD, lung fibrosis, and lung cancer.

UFPs deposit readily in the airways and centriacinar regions of the lung and induce and incite airway diseases such as asthma and COPD and respiratory diseases. Oxidant-mediated cellular damage^4,7^, including the production of reactive oxygen species (ROS) and oxidative stress, innate immunity, and adaptive immunity (Fig. 1), can lead to PM-mediated adverse health effects.

## Reactive oxygen species and oxidative stress

Oxidative stress is highly implicated in the pathogenesis of respiratory diseases. Reactive radical species are ubiquitous in nature and are produced by endogenous and exogenous sources^8^. Cellular organelles such as mitochondria and peroxisomes are major sources of ROS and nitrogen species^9^. Production of reactive species by exogenous sources such as environmental toxins and diet promotes the onset of lung diseases^10^. The physical characteristics and the chemical composition of PM play a key role in ROS generation in vitro and in vivo^8–10^.

Oxygen is readily reduced by an electron to form oxygen free radicals, such as superoxides^11^. In the presence of iron ions, superoxide acquires a second electron, leading to hydrogen peroxide formation, which generated the extremely reactive hydroxyl radical. Hydroxyl radicals react very quickly with biomolecules, such as proteins, fatty acids, and DNA^12–14^. All molecules in the direct vicinity of the hydroxyl radical will react with this reactive form of oxygen^12–15^.

Diesel exhaust particles (DEPs) consist of polyaromatic hydrocarbons, which are hydrophobic molecules that can diffuse easily through cell membranes. As free radicals cause oxidative damage to biological macromolecules, such as DNA, lipids, and proteins, they are believed to be involved in the pathogenesis of many diseases^16^. The particles induce the generation of free radicals, which may lead to an increase in oxidative stress, exacerbating some respiratory symptoms. Metals present on the particle surface, including Fe, Co, Cr, and V, undergo redox cycling, while Cd, Hg, and Ni, as well as Pb, deplete glutathione and protein-bound sulfhydryl groups, resulting in ROS production^17–20^.

PM_10_ exposure at any time during pregnancy is positively associated with levels of mitochondrial 8-hydroxy-2′-deoxyguanosine in maternal blood and umbilical cord blood^21^. PM induces increased mitochondrial oxidative DNA damage during pregnancy in both mothers and their newborns, indicating that particulate air pollution exposure in early life plays a role in increasing systemic oxidative stress at the mitochondrial level, both in the mother and fetus.

The water-insoluble fraction of PM_10_ is similar to the water-soluble fraction of PM_10_ and is also capable of inducing oxidative stress by inducing the generation of hydrogen peroxide and impairing enzymatic antioxidant defense, resulting in oxidative DNA damage and apoptotic cell death through the iron-catalyzed Fenton reaction^22^.

Redox reactions regulate signal transduction as important chemical processes. The response of a cell to a reactive oxygen-rich environment often involves the activation of numerous intracellular signaling pathways, which cause transcriptional changes and allow the cells to respond appropriately to the perceived oxidative stress^13,14^. Nuclear factor-κB (NF-κB), activation protein-1 (AP-1), nuclear factor erythroid 2 related factor 2 (Nrf2), and CREB-binding proteins (CBPs) are regulated and influenced by redox status and have been implicated in the transcriptional regulation of a wide range of genes that are involved in oxidative stress and cellular response mechanisms^23^.

Nrf2^24^ is a major contributor to cellular defense against oxidative damage. There was a significant decrease in the expression of Nrf2 and its upstream regulator genes upon PM_10_ exposure, suggesting that Nrf2 is involved in PM_10_-induced oxidative damage^24^.

Redox status in the nucleus affects histone acetylation and deacetylation status, which regulates inflammatory gene expression by activation of redox-sensitive transcription factors^25^. NF-κB is activated in epithelial cells and inflammatory cells during oxidative stress, leading to the upregulation of many proinflammatory genes^23^. NF-κB is a protein heterodimer that consists of p65 and p50 subunits. NF-κB acts as an inflammatory switch that induces genome-wide epigenetic modification upon ultrafine PM exposure^26^. Many inflammatory genes related to the pathogenesis of asthma are regulated by NF-κB^26^.

AP-1 is a protein dimer composed of a heterodimer of Fos and Jun proteins. AP-1 regulates many of the inflammatory and immune genes in oxidant-mediated diseases. Gene expression of gamma-glutamylcysteine synthetase, the rate-limiting enzyme for GSH synthesis, is induced by activation of AP-1. In addition, the family of mitogen-activated protein kinases is directly or indirectly altered by redox changes^27^. Oxidative stress and other stimuli, such as cytokines, activate various signal transduction pathways, leading to the activation of transcription factors, such as NF-kB and AP-1^28^.

Binding of transcription factors to DNA elements leads to the recruitment of CBP and/or other coactivators to the transcriptional initiation complex on the promoter regions of various genes^28^. Activation of CBP leads to acetylation of specific core histone lysine residues by intrinsic histone acetyltransferase activity^28–30^.

ROS influence airway cells and reproduce many of the pathophysiological features associated with asthma. ROS initiate lipid peroxidation, alter protein structure, enhance the release of arachidonic acid from cell membranes, increase the synthesis and release of chemoattractants, and induce the release of tachykinins and neurokinins^14,15^. This, in turn, augments airway smooth muscle contraction, increases airway reactivity and airway secretions, increases vascular permeability, decreases cholinesterase and neutral endopeptidase activities, and impairs the responsiveness of β-adrenergic receptors^31^.

Asthma attacks are associated with the immediate formation of superoxide that persists throughout the late asthmatic response^32^. Allergen challenge in the airways of atopic individuals causes a twofold increase in superoxide generation^32^. Spontaneous and experimental allergen-induced asthma attacks lead to eosinophil and neutrophil activation, during which NADPH oxidase is activated and ROS, such as superoxide and its dismutation product H_2_O_2,_ are rapidly formed^33^. ROS production in people with asthma correlates with the severity of airway reactivity^34^. Asthma is characterized by oxidative modifications^35^. Increased levels of eosinophil peroxidase (EPO) and myeloperoxidase (MPO) parallel the numbers of eosinophils and neutrophils, respectively, and are found at higher than normal levels in peripheral blood, induced sputum and BAL fluid^36^ of patients with asthma. Malondialdehyde and thiobarbituric acid-reactive substances have also been detected in urine, plasma, sputum, and BAL fluid in relation to the severity of asthma^37,38^. In addition, 8-isoprostane, a biomarker of lipid peroxidation, is also elevated in exhaled breath condensate from adults and children with asthma^37,38^.

Reduced exposure to PM_10_ attenuates age-related declines in lung function, particularly in the small airways^39^. Polymorphisms in glutathione S-transferase (GST) and heme oxygenase-1 (HMOX1) genes, which are important for oxidative stress defense, modify these beneficial effects^39^. A population-based sample of 4365 adults was followed up after 11 years, including questionnaires, spirometry and DNA blood sampling. The benefits of reduced PM_10_ exposure were not equally distributed across the population but were modified by the individual genetic make-up determining oxidative stress defense^39^.

The generation of ROS and nitrogen species is markedly increased during acute asthma attacks^40,41^. Nitric oxide (NO) is a short-lived molecule that causes vasodilation and bronchodilation^42^. In that study, the nitrite concentration in BAL fluid, which is indicative of in vivo generation of NO in the airways, was significantly higher in DEP-exposed animals than in the control group. In another study, alveolar macrophages produced nitrite during in vitro exposure to DEPs (50 μg/ml), with maximal induction 4 h after exposure^43^.

The loss of superoxide dismutase (SOD) contributes to oxidative stress during acute episodes of asthma exacerbation^40,41^. Oxidative modification of manganese SOD (MnSOD) is present in asthmatic airway epithelial cells^44^. The loss of SOD activity reflects increased oxidative and nitrative stress in asthmatic patients, suggesting that SOD serves as a surrogate marker of oxidative stress and asthma severity^45^.

Catalase catalyzes the decomposition of hydrogen peroxide to water and oxygen, and its activity was found to be 50% lower in BAL fluid obtained from individuals with asthma compared to that of healthy controls^46^. Tyrosine oxidant modifications of catalase occur in asthma, such as chlorination of tyrosine by peroxidase-catalyzed halogenation and oxidative crosslinking of tyrosine to form dityrosine, a product of tyrosyl radicals^46^. The most extensive modification found in asthmatic lungs is tyrosine chlorination, which is 20-fold more extensive than that of tyrosine nitration^47^. In contrast to SOD and catalase, extracellular glutathione peroxidase (GPX) is present at higher than normal levels in the lungs of individuals with asthma^47^. This increase is due to induction of GPX mRNA and protein expression by bronchial epithelial cells in response to increased intracellular or extracellular ROS^47^.

During asthma exacerbation in humans, the levels of serum thioredoxin (TRX1) increase and are inversely correlated with airflow^48^. Cigarette smoke induces increased oxidant burden and causes irreversible changes to the protective antioxidant effects in the airways^48^. The smoke-derived oxidants damage airway epithelial cells, inducing direct injury to membrane lipids, proteins, carbohydrates, and DNA, leading to chronic inflammation^48^. Cigarette smoking delivers and generates oxidative stress within the lungs^49^. These imbalances in oxidant burden and antioxidant capacity have been implicated as important contributing factors in the pathogenesis of COPD^49^. However, smoking also causes the depletion of antioxidants, which further contributes to oxidative tissue damage^49^.

Glutathione S-transferases (GSTs) are a family of enzymes that play an important role in detoxification by catalyzing the conjugation of many hydrophobic and electrophilic compounds to reduced glutathione (l-g-glutamyl-l-cysteinyl-glycine) and participating in antioxidant defense through a number of mechanisms, including the repair of ROS-induced damage and the detoxification of xenobiotics present in air pollutants^50^. Glutathione present in human epithelial lining fluid is a key enzyme that protects the lungs from oxidative stress^51^. Titanium dioxide (TiO_2_) particles activate and deactivate the phosphorylation of several inflammatory proteins in lung epithelial cells, especially the serine and tyrosine phosphorylation of GSTP1, which regulates cell damage and apoptosis following exposure to TiO_2_ particles. Collectively, our data suggest that GSTP1 is an important modulator of TiO_2_ particle-induced inflammation^52^.

The downregulation of antioxidant pathways has also been associated with acute exacerbations of COPD^49^. Disruption of the oxidant/antioxidant balance is important in the pathogenesis of acute lung injury and acute respiratory distress syndrome. Different cytokines and growth factors play a role in the pathogenesis of lung fibrosis^53^. ROS mediate TGF-β formation in lung epithelial cells^53^.

## Innate immunity

Particles larger than 10 μm generally get caught in the nose and throat and never enter the lungs^54,55^. Particles less than 10 μm but greater than 2 μm land in the tracheobronchial tree and are cleared by mucociliary clearance. Smaller particles can transverse through the airways and deposit in the alveolar region. In this region, phagocytic cells, including neutrophils and macrophages, are recruited to foreign particles by cytokines and chemokines and engulf the particles by phagocytosis^54,55^. The mucociliary escalator then transports particle-laden neutrophils and macrophages^56^. PM induces the release of inflammatory cytokines, such as IL-6, IL-8, GM-CSF, and TNF-α^57^, from immune cells (e.g., macrophages) as well as structural airway cells^58,59^.

Chitin is commonly found in organisms including parasites, fungi, and bacteria but does not occur in mammalian tissues^60^, allowing for selective antimicrobial activity of chitinase. Macrophage-synthesized Ym1 and Ym2 are homologous to chitinase and have chitinase activity^61,62^. Through the IL-4/STAT 6 signal transduction pathway, Ym1 is implicated in allergic peritonitis^63^. Acid mammalian chitinase may also be an important mediator of IL-13-induced responses in Th2 disorders, such as asthma^64^. Indeed, polymorphisms in acid mammalian chitinase are associated with asthma, further supporting the involvement of acid mammalian chitinase in asthma development^65^. DEPs induce airway hyperresponsiveness (AHR), as well as Ym mRNA expression, which is a Th2 cell-biased response by activated macrophages^66^. The chitinase Ym1 is expressed in the spleen and lungs, with lower expression in the thymus, intestine, and kidney, whereas Ym2 is expressed at high levels in the stomach, with lower levels in the thymus and kidney^66^. Conserved STAT6 sites probably account for the similar, striking induction of Ym1 and Ym2 expression in Th2-type environments. In a murine model of DEP exposure, BALB/c mice intranasally exposed to DEPs followed by a DEP challenge had upregulation of lung-specific expression of Ym1 and Ym2 transcripts relative to that of mice that were not exposed nor similarly challenged^43^. The regulation and function of chitinase have not been well explored in air pollution asthma models. However, in one study, Ym1 was one of the most highly induced IL-4 target genes, exhibiting at least a 70-fold increase in macrophage populations^43^. Alveolar macrophages play an important role in particle-induced airway and lung inflammation via direct production of IL-13.

**Fig. 2:**
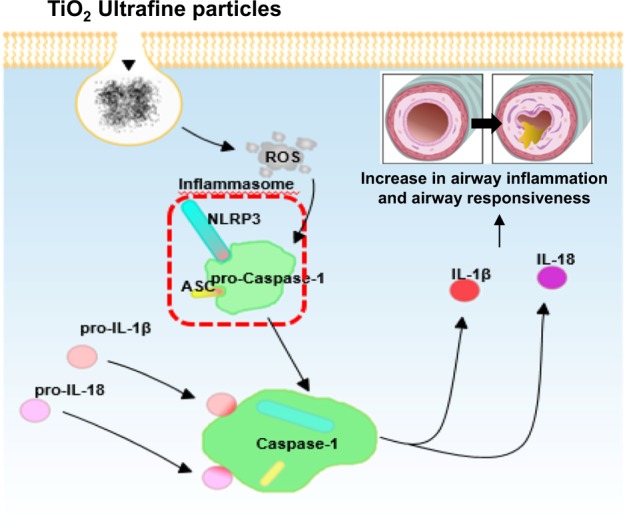
Schematic of the inflammasome cascade in the lungs of the TiO_2_ particle-exposed model.

Proteomics offers a unique means of analyzing expressed proteins and has been successfully used to examine the effects of oxidative stress at the cellular level^67^. In addition to revealing protein modifications, this approach is also used to assess changes in protein expression levels^68^. In a previous study, 20 proteins were identified whose expression levels in the human bronchial epithelial cell line BEAS-2B changed in response to TiO_2_ particle exposure^69^. These proteins included defense-related, cell-activating, and cytoskeletal proteins that are implicated in the response to oxidative stress and can be classified into four groups according to the pattern of the TiO_2_-induced change in expression over time. One protein, macrophage migration inhibitory factor (MIF, Fig. 2), was also induced at the transcriptional level. Similarly, black carbon and diesel exhaust particles induced the protein expression of MIF in BEAS-2B cells. The expression of MIF also increased in the lungs of TiO_2_-instilled rats. These results indicate that a portion of these proteins may serve as mediators of or markers for airway disease caused by exposure to PM.

The inflammatory effects of PM_10_ have been demonstrated in experimental animal studies by using direct instillation into the lung prior to human studies that showed pulmonary effects after experimental exposure to PM^17^. Clinically, PM_10_ particles likely provoke airway inflammation via the release of mediators that exacerbate lung disease in susceptible individuals^70^; even a single exposure compromises a host’s ability to respond to ongoing pulmonary infections^71^. Fine and UFPs directly stimulate macrophages and epithelial cells to produce inflammatory cytokines such as TNF-α, TGF-β1, GM-CSF, PDGF, IL-6, and IL-8^72^, and reactive oxygen species are responsible for acute and chronic lung inflammation^73^.

**Fig. 3:**
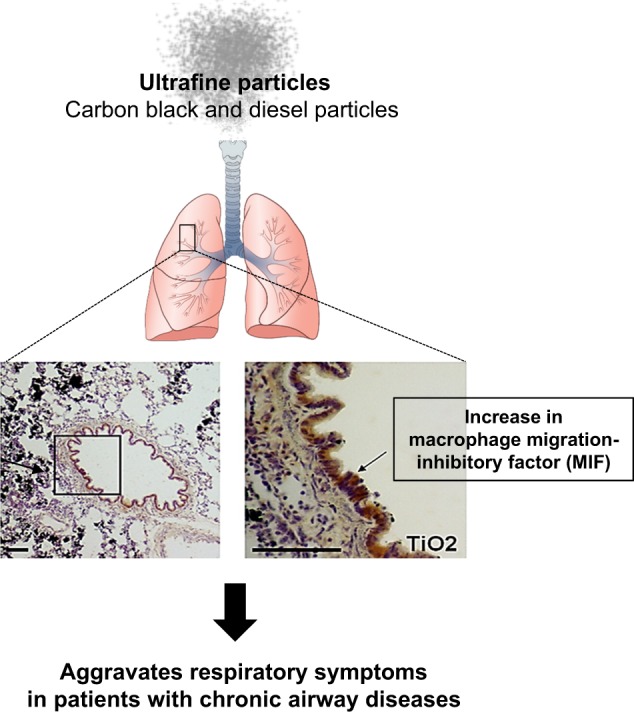
PM exposure initiates innate immunity through macrophage migration inhibitory factors, leading to exacerbation of respiratory symptoms.

The inflammasome is a multiprotein complex that regulates inflammation by activating specific proinflammatory cytokines, resulting in an effective host immune response^74^. The innate immune system is the first line of host defense, and the inflammasome is essential for maintaining a delicate balance between pro- and anti-inflammatory signals to generate an appropriate immune response without harming the host^74^. The inflammasome is a major regulator of inflammation through its activation of pro-caspase-1, which cleaves pro-interleukin-1β (pro-IL-1β) into its mature form. IL-1β is a critical proinflammatory cytokine that controls the severity of inflammation associated with a wide spectrum of inflammatory diseases. NAIP, CIITA, HET-E, TP-2 (NACHT), and leucine-rich repeat and pyrin domain-containing protein 3 (NLRP3) are key components of the inflammasome complex, and multiple signals and stimuli trigger formation of the NLRP3 inflammasome complex^75^. In our studies^76^, AHR and inflammation increased in OVA-sensitized/challenged mice, and these responses were exacerbated by exposure to TiO_2_ particles (Fig. 3). TiO_2_ particle exposure increased IL-1β and IL-18 expression in OVA-sensitized/challenged mice. UFPs augmented the expression of NLRP3 and caspase-1, leading to the production of active caspase-1 in the lung. Caspase-1 expression was increased and exacerbated by exposure to TiO_2_ particles in OVA-sensitized/challenged mice. ROS levels tended to increase in OVA-sensitized/challenged and OVA-sensitized/challenged-plus-TiO_2_ particle-exposed mice. Our data demonstrate that inflammasome activation occurred in asthmatic lungs following exposure to particles, suggesting that targeting the inflammasome may assist in controlling particle-induced airway inflammation and AHR.

The effect of air pollution-related PM on epithelial barrier function and tight junction (TJ) expression in human nasal mucosa has not been studied to date. Exposure to PM_2.5_ leads to a loss of barrier function in the human nasal epithelium through decreased expression of TJ proteins and increased release of proinflammatory cytokines^77^.

## Adaptive immunity

**Fig. 4:**
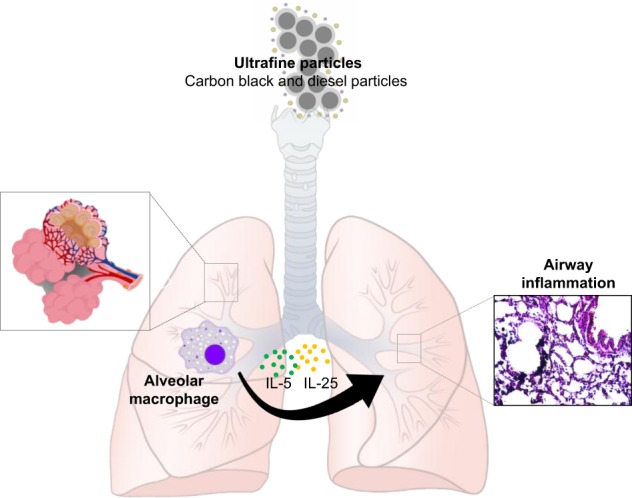
Th2 cytokine changes in macrophages exposed to TiO_2_ particles.

PM causes an increase in changes in T cell responses. PM induces a Th2-like microenvironment in the lung, with overproduction of IL-4 and IL-13^68^. Lung IL-13 transcripts increased 24 h after treatment with fine TiO_2_ particles (mean diameter = 0.29 μm) compared to that of sham-treated rats^68^. IL-13 levels also increased in the BAL fluids of TiO_2_-treated rats 72 h after treatment relative to those of sham-treated rats. To investigate the time- and dose-dependence of macrophage IL-13 production, isolated alveolar macrophages were stimulated with 1, 10, and 40 μg/ml TiO_2_ for 24, 48, and 72 h. The control group consisted of untreated alveolar macrophages. IL-13 levels in the supernatants of the macrophage cultures were measured by ELISA. Macrophages cultured for 48 h with TiO_2_ produced IL-13 in a dose-dependent manner. In addition, 10 μg/ml TiO_2_ significantly enhanced IL-13 production relative to that of the controls. IL-13 protein production increased in a time-dependent manner and peaked 48 h after TiO_2_ exposure. Using immunohistochemical staining, we also found that macrophages that were engulfing TiO_2_ were the main source of IL-13 in TiO_2_ particle-induced lung inflammation. Taken together, our results suggest that alveolar macrophages are major effectors of innate immunity by modulating inflammatory responses towards a Th2 phenotype by producing IL-13, as seen in the adaptive immune response (Fig. 4).

Currently, evidence is not sufficient to demonstrate a direct relationship between particulates and the induction of Th2-like cytokines, including IL-4 and IL-13. TiO_2_ particles are a component of PM_10_ found in dusty workplaces in industries that are involved in the crushing and grinding of the mineral ore rutile^78^, and 50% of TiO_2_-exposed workers have respiratory symptoms accompanied by reduced pulmonary function. Because acute and chronic exposure to TiO_2_ particles also induce inflammatory responses in the airways and alveolar spaces of rats^68,79–81^, TiO_2_-treated rats are a useful model for studying epithelial responses to PM_10_ particles.

PM_10_ or DEPs increase lung inflammation by inhaled allergens or respiratory viral infection by acting as adjuvants. The response may enhance existing allergies or IgE responses to neo-allergens and susceptibility to respiratory infection. This adjuvant effect is exerted by the enhanced production of inflammatory Th2 and/or Th1 cytokines^59^. In animal experiments and human studies, several cytokines and CC chemokines, including IL-4, IL-5, IL-13, GM-CSF, RANTES, MCP-3, and MIP-1, were increased when lymphocytes and macrophages/monocytes were costimulated with particulates in the presence of specific allergens^82^. The immune system responds in different ways depending on the type of particulate. DEPs favor a Th2 response, while asbestos fiber and carbon particles upregulate both Th1 and Th2 cytokines produced by autologous lymphocytes stimulated by antigen^82^.

In addition to adjuvant effects, inhaled inert particles cause a spectrum of pulmonary responses, ranging from minimal changes to marked acute and chronic inflammation. In our study, BALB/c mice were exposed to 100 μg/m³ (low dose) or 3 mg/m³ (high dose) DEPs for up to 12 weeks (1 h/d × 5 d/wk)^83^. AHR increased more in the DEP group than in the control group, and increased more in the high-dose DEP group than in the low-dose DEP group at 4, 8, and 12 weeks. IL-5, IL-13, and interferon-γ increased more in the low-dose DEP group than in the control group at 12 weeks. IL-10 was higher in the high-dose DEP group than in the control group at 12 weeks. Vascular endothelial growth factor was increased in the low-dose and high-dose DEP groups compared to that of the control group at 12 weeks. Transforming growth factor-β increased more in the high-dose DEP group than in the control group at 4, 8, and 12 weeks. The lung collagen content and lung fibrosis were increased in the high-dose DEP group at 8 and 12 weeks. These results suggest that long-term DEP exposure increases AHR, inflammation, lung fibrosis, and goblet cell hyperplasia in a mouse model.

## Other mechanisms

**Fig. 5:**
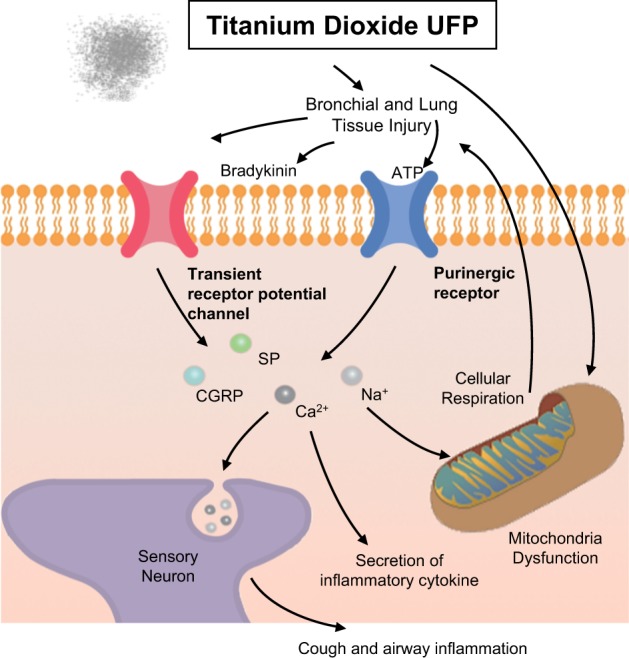
PM exposure triggers neuroinflammation causing cough and airway inflammation.

Neurogenic inflammation in the lung involves airway obstruction, an increase in vascular permeability, extravasation of plasma and leukocytes, mucus hypersecretion and the release of additional inflammatory mediators^84^. The neurogenic inflammatory pathway is associated with the release and activity of neuropeptides such as tachykinins and calcitonin gene-related peptide as a response of sensory neurons to inflammatory mediators and noxious stimuli^84,85^. Transient receptor potential vanilloid 1 (TRPV1) plays a particularly important role in increasing C-fiber excitability and neuronal inflammatory pathways during airway inflammation^86^. ATP and histamine responses to tussive stimuli are activated via P2X receptor-mediated mechanisms^87,88^. P2X7 receptors, which play a role in neuroinflammation, are frequently coexpressed with another P2X receptor, P2X4^89^. Silica nanoparticles inhibit TRPV4 activation and impair the positive modulatory action of TRPV4 channel stimulation on the frequency of ciliary beating in airway epithelial cells^90^. The P2X7 receptor is involved in inflammation triggered by SiO_2_ and TiO_2_ UFPs by increasing IL-1β secretion, likely through the inflammasome pathway^91^. In our study^92^, bradykinin, ATP, substance P and CGRP levels in BALF were increased in OVA mice, and these increases were augmented in OVA plus UFP-exposed mice and in NHBE cells with increasing UFP doses, suggesting that UFPs activate TRPVs and P2X7 and secrete neuromediators that lead to airway inflammation, exacerbating asthma. Our data^92^ revealed that TRPV1, TRPV4, P2X4, and P2X7 were involved in the pathogenesis of bronchial asthma and that UFPs exacerbate asthma via a neurogenic mechanism (Fig. 5).

## Conclusions

Human and animal studies suggest that PM is involved in the pathogenesis of airway inflammation and exacerbates respiratory diseases. The mechanism of UFP-induced human health effects can be explained by oxidative cellular damage, including innate immunity, adaptive immunity, and reactive oxygen species. Further studies are needed to clarify the mechanism by which UFPs induce health effects to prevent respiratory and human diseases by UFPs.

## References

[1] Schäfer T, Ring J (1997). Epidemiology of allergic diseases. Allergy.

[2] Surawski NC (2011). Physicochemical characterization of particulate emissions from a compression ignition engine: the influence of biodiesel feedstock. Environ. Sci. Technol..

[3] McCormack MC (2008). Common household activities are associated with elevated particulate matter concentrations in bedrooms of inner-city Baltimore pre-school children. Environ. Res..

[4] Nel A, Xia T, Madler L, Li N (2006). Toxic potential of materials at the nanolevel. Science.

[5] Hopke PK (2019). Changes in the acute response of respiratory diseases to PM2.5 in New York State from 2005 to 2016. Sci. Total Environ..

[6] Doiron D (2019). Air pollution, lung function and COPD: results from the population-based UK Biobank study. Eur. Respir. J..

[7] Jang, A. S. in *Air Pollution: A Comprehensive Perspective*. (eds Haryanto B.) 153–174 (Intech, Rijeka, 2012).

[8] Kang J, Pervaiz S (2012). Mitochondria: redox metabolism and dysfunction. Biochem. Res. Int..

[9] Fransen M, Nordgren M, Wang B, Apanasets O (2012). Role of peroxisomes in ROS/RNS-metabolism: implications for human disease. Biochim. Biophys. Acta.

[10] Villegas L, Stidham T, Nozik-Grayck E (2014). Oxidative stress and therapeutic development in lung diseases. J. Pulm. Respir. Med..

[11] Bast A, Weseler AR, Haenen GR, den Hartog GJ (2010). Oxidative stress and antioxidants in interstitial lung disease. Curr. Opin. Pulm. Med..

[12] Finkel T (2011). Signal transduction by reactive oxygen species. J. Cell. Biol..

[13] Comhair SA, Erzurum SC (2010). Redox control of asthma: molecular mechanisms and therapeutic opportunities. Antioxid. Redox Signal..

[14] Nadeem A, Masood A, Siddiqui N (2008). Oxidant–antioxidant imbalance in asthma: scientific evidence, epidemiological data and possible therapeutic options. Ther. Adv. Respir. Dis..

[15] Trédaniel J, Boffetta P, Saracci R, Hirsch A (1994). Exposure to environmental tobacco smoke and adult non-neoplastic respiratory diseases. Eur. Respir. J..

[16] Stohs SJ, Bagci D, Hassoun E, Bagchi M (2001). Oxidative mechanisms in the toxicity of chromium and cadmium ions. J. Environ. Pathol. Toxicol. Oncol..

[17] Ghio AJ, Devlin RB (2001). Inflammatory lung injury after bronchial instillation of air pollution particles. Am. J. Respir. Crit. Care Med..

[18] Valko M, Morris H, Cronin MT (2005). Metals, toxicity and oxidative stress. Curr. Med. Chem..

[19] Forman HJ, Torres M (2001). Redox signaling in macrophages. Mol. Asp. Med..

[20] Beamer CA, Holian A (2005). Scavenger receptor class A type I/II (CD204) null mice fail to develop fibrosis following silica exposure. Am. J. Physiol. Lung Cell. Mol. Physiol..

[21] Grevendonk L (2016). Mitochondrial oxidative DNA damage and exposure to particulate air pollution in mother-newborn pairs. Environ. Health.

[22] Yi S, Zhang F, Qu F, Ding W (2014). Water-insoluble fraction of airborne particulate matter (PM10) induces oxidative stress in human lung epithelial A549 cells. Environ. Toxicol..

[23] Bhargava A (2019). Exposure to ultrafine particulate matter induces NF-κβ mediated epigenetic modifications. Environ. Pollut..

[24] Radan M (2019). n vivo and in vitro evidence for the involvement of Nrf2-antioxidant response element signaling pathway in the inflammation and oxidative stress induced by particulate matter (PM10): the effective role of gallic acid. Free Radic. Res..

[25] Gambhir L, Sharma V, Kandwal P, Saxena S (2019). Perturbation in cellular redox homeostasis: decisive regulator of T cell mediated immune responses. Int. Immunopharmacol..

[26] Ciencewicki J, Trivedi S, Kleeberger SR (2008). Oxidants and the pathogenesis of lung diseases. J. Allergy Clin. Immunol..

[27] Rahman I, Adcock IM (2006). Oxidative stress and redox regulation of lung inflammation in COPD. Eur. Respir. J..

[28] Carvalho H, Evelson P, Sigaud S, González-Flecha B (2004). Mitogen-activated protein kinases modulate H(2)O(2)-induced apoptosis in primary rat alveolar epithelial cells. J. Cell. Biochem..

[29] Nguyen T, Sherratt PJ, Pickett CB (2003). Regulatory mechanisms controlling gene expression mediated by the antioxidant response element. Annu. Rev. Pharmacol. Toxicol..

[30] Barnes PJ, Chung KF, Page CP (1998). Inflammatory mediators of asthma: an update. Pharmacol. Rev..

[31] Calhun WJ, Reed HE, Moest DR, Stevens CA (1992). Enhanced superoxide production by alveolar macrophages and air-space cells, airway inflammation, and alveolar macrophage density changes after segmental antigen bronchoprovocation in allergic subjects. Am. Rev. Respir. Dis..

[32] Klebanoff SJ (1980). Oxygen metabolism and the toxic properties of phagocytes. Ann. Intern. Med..

[33] Sanders SP (1995). Spontaneous oxygen radical production at sites of antigen challenge in allergic subjects. Am. J. Respir. Crit. Care Med..

[34] Mondino C (2004). Effects of inhaled corticosteroids on exhaled leukotrienes and prostanoids in asthmatic children. J. Allergy Clin. Immunol..

[35] Wood LG (2005). Induced sputum 8-isoprostane concentrations in inflammatory airway diseases. Am. J. Respir. Crit. Care Med..

[36] MacPherson JC (2001). Eosinophils are a major source of nitric oxide-derived oxidants in severe asthma: characterization of pathways available to eosinophils for generating reactive nitrogen species. J. Immunol..

[37] Wu W (2000). Eosinophils generate brominating oxidants in allergen-induced asthma. J. Clin. Invest..

[38] Malik AI, Storey KB (2011). Transcriptional regulation of antioxidant enzymes by FoxO1 under dehydration stress. Gene.

[39] Curjuric I (2010). HMOX1 and GST variants modify attenuation of FEF25-75% decline due to PM10 reduction. Eur. Respir. J..

[40] Takaku Y (2011). IFN-γ-inducible protein of 10 kDa upregulates the effector functions of eosinophils through β2 integrin and CXCR3. Respir. Res..

[41] Kuroki M (1996). Reactive oxygen intermediates increase vascular endothelial growth factor expression in vitro and in vivo. J. Clin. Invest..

[42] Moncada S, Palmer RM, Higgs EA (1991). Nitric oxide: physiology, pathophysiology and pharmacology. Pharmacol. Rev..

[43] Song HM (2008). Ym1 and Ym2 expression in a mouse model exposed to diesel exhaust particles. Environ. Toxicol..

[44] Ghosh S (2003). Nitration of proteins in murine model of asthma. Am. J. Respir. Crit. Care Med..

[45] Comhair SA, Erzurum SC (2002). Antioxidant responses to oxidant-mediated lung diseases. Am. J. Physiol. Lung Cell. Mol. Physiol..

[46] Yamada Y (2003). Elevated serum levels of thioredoxin in patients with acute exacerbation of asthma. Immunol. Lett..

[47] van der Toorn M (2007). Cigarette smoke-induced blockade of the mitochondrial respiratory chain switches lung epithelial cell apoptosis into necrosis. Am. J. Physiol. Lung Cell. Mol. Physiol..

[48] Foronjy R, Alison W, D’Aarmiento J (2008). The pharmokinetic limitations of antioxidant treatment for COPD. Pulm. Pharmacol. Ther..

[49] Lin JL, Thomas PS (2010). Current perspectives of oxidative stress and its measurement in chronic obstructive pulmonary disease. COPD.

[50] Strange RC, Jones PW, Fryer AA (2000). Glutathione S-transferase: genetics and role in toxicology. Toxicol. Lett..

[51] Cantin AM, North SL, Hubbard RC, Crystal RG (1987). 1987. Normal alveolar epithelial lining fluid contains high levels of glutathione. J. Appl. Physiol..

[52] Kim TH (2011). Particle stimulation dephosphorylates glutathione S-transferase π1 of epithelial cells. Toxicology.

[53] Hecker L (2009). NADPH oxidase-4 mediates myofibroblasts activation and fibrogenic responses to lung injury. Nat. Med..

[54] Seagrave J (2008). Mechanisms and implications of air pollution particle associations with chemokines. Toxicol. Appl. Pharmacol..

[55] Yang W, Omaye ST (2009). Air pollutants, oxidative stress and human health. Mutat. Res..

[56] Donaldson K, Tran CL (2002). Inflammation caused by particles and fibers. Inhal. Toxicol..

[57] Stone V, Johnston H, Clift MJD (2007). Air pollution, ultrafine and nanoparticle toxicology: cellular and molecular interactions. IEEE Trans. Nanobiosci..

[58] Totlandsdal AI, Cassee FR, Schwarze P, Refsnes M, Låg M (2010). Diesel exhaust particles induce CYP1A1 and pro-inflammatory responses via differential pathways in human bronchial epithelial cells. Part. Fibre Toxicol..

[59] Diaz-Sanchez D, Tsien A, Fleming J, Saxon A (1997). Combined diesel exhaust particulate and ragweed allergen challenge markedly enhances human in vivo nasal ragweed-specific IgE and skews cytokine production to a T helper cell 2-type pattern. J. Immunol..

[60] Guo L, Johnson RS, Schuh JC (2000). Biochemical characterization of endogenously formed eosinophilic crystals in the lungs of mice. J. Biol. Chem..

[61] Sun YJ (2001). The crystal structure of a novel mammalian lectin, Ym1, suggests a saccharide binding site. J. Biol. Chem..

[62] Jin HM (1998). Genetic characterization of the murine Ym1 gene and identification of a cluster of highly homologous genes. Genomics.

[63] Welch JS (2002). TH2 cytokines and allergic challenge induce Ym1 expression in macrophages by a STAT6-dependent mechanism. J. Biol. Chem..

[64] Zhu Z (2004). Acidic mammalian chitinase in asthmatic Th2 inflammation and IL-13 pathway activation. Science.

[65] Bierbaum S (2005). Polymorphisms and haplotypes of acid mammalian chitinase are associated with bronchial asthma. Am. J. Respir. Crit. Care Med..

[66] Ward JM (2001). Hyalinosis and Ym1/Ym2 gene expression in the stomach and respiratory tract of 129S4/SvJae and wild-type and CYP1A2-null B6, 129 mice. Am. J. Pathol..

[67] Xiao GG, Wang M, Li N, Loo JA, Nel AE (2003). Use of proteomics to demonstrate a hierarchical oxidative stress response to diesel exhaust particle chemicals in a macrophage cell line. J. Biol. Chem..

[68] Kang CM (2005). Interleukin-25 and interleukin-13 production by alveolar macrophages in response to particles. Am. J. Respir. Cell Mol. Biol..

[69] Cha MH (2007). Proteomic identification of macrophage migration-inhibitory factor upon exposure to TiO_2_ particles. Mol. Cell. Proteom..

[70] Seaton A, MacNee W, Donaldson K, Godden D (1995). Particulate airpollution and acute health effects. Lancet.

[71] Zelikoff JT (2003). Effects of inhaled ambient particulate matter on pulmonary antimicrobial immune defense. Inhal. Toxicol..

[72] Fujii T, Hayashi S, Hogg JC, Vincent R, Van Eeden SF (2001). Particulate matter induces cytokine expression in human bronchial epithelial cells. Am. J. Respir. Cell Mol. Biol..

[73] Liu H, Colavitti R, Rovira II, Finkel T (2005). Redox-dependent transcriptional regulation. Circ. Res..

[74] Abais JM, Xia M, Zhang Y, Boini KM, Li PL (2015). Redox regulation of NLRP3 inflammasomes: ROS as trigger or effector?. Antioxid. Redox Signal..

[75] Bose S (2014). ADP-ribosylation of NLRP3 by mycoplasma pneumoniae CARDS toxin regulates inflammasome activity. MBio..

[76] Kim BG, Lee PH, Lee SH, Park MK, Jang AS (2017). Effect of TiO_2_ nanoparticles on inflammasome-mediated airway inflammation and responsiveness. Allergy Asthma Immunol. Res..

[77] Xian M (2020). Particulate matter 2.5 causes deficiency in barrier integrity in human nasal epithelial cells. Allergy Asthma Immunol. Res.

[78] Templeton, D. M. in *Handbook On Metals In Clinical And Analytic Chemistry* (eds Seiler H. G., Siegel A., Siegel H.) 627–630 (Marcel Dekker, New York, 1994).

[79] Ahn MH (2005). Titanium dioxide particle-induced goblet cell hyperplasia: association with mast cells and IL-13. Respir. Res..

[80] Schapira RM (1995). Hydroxyl radical production and lung injury in the rat following silica or titanium dioxide instillation in vivo. Am. J. Respir. Cell Mol. Biol..

[81] Warheit DB (1997). Inhalation of high concentrations of low toxicity dusts in rats results in impaired pulmonary clearance mechanisms and persistent inflammation. Toxicol. Appl. Pharmacol..

[82] Hamilton RF, Holian A, Morandi MT (2004). A comparison of asbestos and urban particulate matter in the in vitro modification of human alveolar macrophage antigen-presenting cell function. Exp. Lung Res..

[83] Kim BG (2016). Long-term effects of diesel exhaust particles on airway inflammation and remodeling in a mouse model. Allergy Asthma Immunol. Res..

[84] Butler CA, Heaney L (2007). Neurogenic inflammation and asthma. Inflamm. Allergy Drug Targets.

[85] Banner KH, Igney F, Poll C (2011). TRP channels: emerging targets for respiratory disease. Pharmacol. Ther..

[86] Colsoul B, Nilius B, Vennekens R (2009). On the putative role of transient receptor potential cation channels in asthma. Clin. Exp. Allergy.

[87] Jacob F, Pérez Novo C, Bachert C, Van CK (2013). Purinergic signaling in inflammatory cells: P2 receptor expression, functional effects, and modulation of inflammatory responses. Purinergic Signal..

[88] Monção-Ribeiro (2014). P2X7 receptor modulates inflammatory and functional pulmonary changes induced by silica. PLoS ONE.

[89] Abdulqawi R (2015). P2X3 receptor antagonist (AF-219) in refractory chronic cough: a randomised, double-blind, placebo-controlled phase 2 study. Lancet.

[90] Sanchez A (2017). Silica nanoparticles inhibit the cation channel TRPV4 in airway epithelial cells. Part. Fibre Toxicol..

[91] Dekali S (2013). Cell cooperation and role of the P2X_7_ receptor in pulmonary inflammation induced by nanoparticles. Nanotoxicology.

[92] Kim BG (2020). Effects of nanoparticles on neuroinflammation in a mouse model of asthma. Respir. Physiol. Neurobiol..

